# Current Models of Mammalian Target of Rapamycin Complex 1 (mTORC1) Activation by Growth Factors and Amino Acids

**DOI:** 10.3390/ijms151120753

**Published:** 2014-11-13

**Authors:** Xu Zheng, Yan Liang, Qiburi He, Ruiyuan Yao, Wenlei Bao, Lili Bao, Yanfeng Wang, Zhigang Wang

**Affiliations:** 1 College of Life Sciences, Inner Mongolia University, Hohhot 010021, China; E-Mails: dong2186@163.com (X.Z.); xixils@163.com (Y.L.); qbr2010@126.com (Q.H.); 18647808043@163.com (R.Y.); bwlnhm@sina.com (W.B.); baolili1203@126.com (L.B.); 2 Department of Clinical Laboratory, Hulunbeir Municipal People’s Hospital, Hailar 021008, China; 3 College of Basic Medical Science, Inner Mongolia Medical University, Hohhot 010110, China

**Keywords:** mTORC1, Rheb, Ragulator, Rag GTPases, hVps34, PA

## Abstract

Mammalian target of rapamycin (mTOR), which is now referred to as mechanistic target of rapamycin, integrates many signals, including those from growth factors, energy status, stress, and amino acids, to regulate cell growth and proliferation, protein synthesis, protein degradation, and other physiological and biochemical processes. The mTOR-Rheb-TSC-TBC complex co-localizes to the lysosome and the phosphorylation of TSC-TBC effects the dissociation of the complex from the lysosome and activates Rheb. GTP-bound Rheb potentiates the catalytic activity of mTORC1. Under conditions with growth factors and amino acids, v-ATPase, Ragulator, Rag GTPase, Rheb, hVps34, PLD1, and PA have important but disparate effects on mTORC1 activation. In this review, we introduce five models of mTORC1 activation by growth factors and amino acids to provide a comprehensive theoretical foundation for future research.

## 1. Introduction

TOR (target of rapamycin) is an evolutionarily highly conserved serine/threonine kinase that belongs to the phosphatidylinositol kinase-related kinase (PIKK) family [1,2]. Shortly after its identification in *Saccharomyces cerevisiae*, a mammalian ortholog of TOR was identified and named FK506-binding protein 12 (FABP12)-rapamycin-associated protein (FRAP), or rapamycin and FKBP12 target (RAFT). Later, it was officially named mammalian target of rapamycin (mTOR) [3,4,5,6,7]. Recently, mTOR was renamed mechanistic target of rapamycin (MTOR) by the HUGO Gene Nomenclature Committee (HGNC). Rapamycin, a specific inhibitor of mTOR, inhibits cell growth and proliferation [5,8,9].

mTOR combines with various components to form two mTOR complexes, mTORC1 and mTORC2. mTORC1 comprises mTOR, Raptor (regulator-associated protein of mTOR), PRAS40 (proline-rich Akt substrate, 40 kDa), Deptor, mLst8 (mammalian lethal with SEC13 protein 8), Tti1, and Tel2. mTORC2 is composed of mTOR, Rictor, mSin1 (mammalian stress-activated protein kinase-interacting protein 1), Protor1/2, Deptor, mLst8, Tti1, and Tel2. Raptor in mTORC1 and Rictor in mTORC2 have equivalent significance. The phosphorylation or acetylation of Rictor influences generation of mTORC2 [10,11,12]. Raptor and PRAS40 are unique to mTORC1, as Rictor, mSin1, and Protor1/2 are to mTORC2; the difference between Raptor and Rictor is likely the basis for their disparate functions.

The consensus over the past several decades is that mTORC1 is sensitive to rapamycin, whereas mTORC2 is rapamycin insensitive, although recent reports indicate that prolonged rapamycin treatment prevents the *de novo* generation of mTORC2 [8,13,14,15,16,17]. Consequently, mTORC1 has been studied in greater detail, due to its sensitivity to rapamycin.

The six components of mTORC1 have varying influences on mTORC1 signaling. Raptor, mLST8, Tti1, and Tel2 are positive regulators, whereas PRAS40 and Deptor are negative regulators. Raptor interacts with mTOR at multiple points and binds to downstream effectors of mTOR, including S6K1 (ribosomal protein S6 kinase 1) and 4E-BP1 (eukaryotic translation initiation factor 4E-binding protein 1), which are the chief downstream factors in protein synthesis and ribosome formation [18,19,20,21,22,23]. S6K1 is a kinase of S6, which controls protein synthesis, and 4E-BP1 governs the release of the translation initiation factor eIF4E [1,24,25]. 

In addition, ULK1 (unc-51-like autophagy activating kinase 1), a homolog of yeast ATG1, is believed to be another downstream effector of mTORC1. ULK1 is a key initiator of mammalian autophagy, and its activation is prevented by mTORC1 under nutrient sufficiency [26]. mLST8 interacts specifically with the kinase domain of mTOR and regulates the stability of mTOR-Raptor association under various nutritional conditions [18,27]. mLST8 receives upstream signals, including nutrient factors and growth factors, and overexpression of mLST8 stimulates mTOR kinase activity [18,27]. However, deletion of mLST8 does not affect mTORC1 activity in mice [28,29]. Tti1, a putative novel mTOR-binding protein, positively regulates mTOR activity and interacts with Tel2, a common partner in the assembly of the mTOR complex, to maintain its activity [30,31]. Raptor, mLTS8, Tti1, and Tel2 are essential for activating mTORC1 signaling by growth factors and amino acids.

PRAS40 was isolated as a substrate of AKT (also known as protein kinase B, PKB) [32]. The phosphorylation of PRAS40 leads to the interaction between it and 14-3-3 protein, which associates with transcription factors, signaling molecules, apoptosis factors, and tumor suppressors to promote cell survival as a phosphoserine-/phosphothreonine-binding protein [32,33]. PRAS40 is phosphorylated by mTOR. By binding to mTORC1 via Raptor, PRAS40 inhibits mTORC1 autophosphorylation and mTORC1 kinase activity toward 4EBP1 and itself [34,35]. Knockdown of PRAS40 impairs the amino acid- and insulin-stimulated phosphorylation of 4EBP1 and S6K1, demonstrating that PRAS40 is required for signaling downstream of mTORC1 [36,37,38]. 

Deptor, an mTOR inhibitor, is also negatively regulated by mTOR. Knockdown of Deptor ameliorates disuse muscle atrophy [39,40,41]. Deptor phosphorylation by mTOR in response to growth signals cooperates with casein kinase I (CK I) to generate a degron that binds to an F box protein, βTrCP, for subsequent ubiquitination and degradation [42,43,44]. Consequently, mTOR generates an autoamplification loop to stimulate its activity. 

## 2. Model of mTORC1 Activation by Growth Factors

Growth factors, such as insulin and insulin-like growth factors (IGFs), stimulate mTORC1 through the PI3K (phosphatidylinositol-4,5-bisphosphate 3-kinase) and Ras signaling pathways [2,29,45], which enhances the phosphorylation of the TSC-TBC complex by protein kinase B (AKT), extracellular signal-regulated kinase 1/2 (ERK1/2), and p90 ribosomal S6 kinase (RSK1) [29,46,47,48,49,50]. The TSC-TBC complex, also known as Rhebulator, comprises TSC1 (tuberous sclerosis 1), TSC2 (tuberous sclerosis 2), and TBC1D7 (TBC1 domain family, member 7) [46,51]. TSC2 is a GTPase-activating protein (GAP) that induces GTP hydrolysis of Rheb (Ras homolog enriched in brain), which is a Ras-like small guanosine triphosphatase (GTPase) that shuttles between an active GTP-bound form and an inactive GDP-bound form [52,53]. Thus, TSC2 hydrolyzes Rheb-GTP to Rheb-GDP and in turn inhibits mTORC1 kinase activity [54].

**Figure 1:**
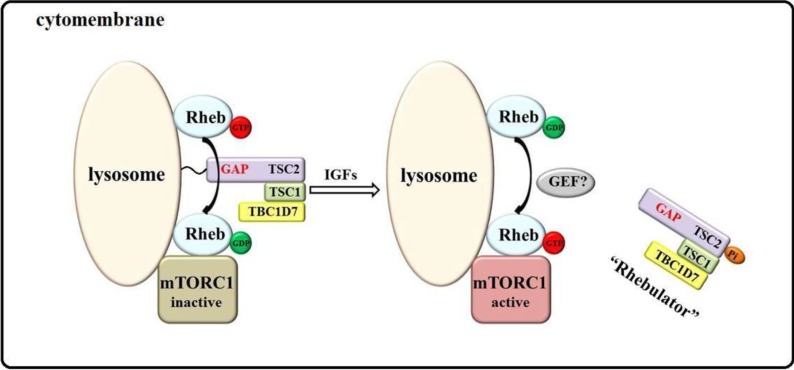
The Rhebulator-Rheb model of mTORC1 activation by growth factors. mTOR, Rheb, and the TSC-TBC complex colocalize on the lysosomal surface, and the GAP activity of TSC2 inhibits the function of GTP-bound Rheb. Growth factors induce the phosphorylation of the TSC-TBC complex, resulting in its dissociation from the lysosome, although TSC2 continues to have GAP activity. This separation of the TSC-TBC complex prevents TSC2 from interacting with Rheb-GTP on the lysosome to inhibit its activity. Under the effects of GEFs or its own activity, GDP-bound Rheb converts into GTP-bound Rheb, stimulating the kinase activity of mTORC1.

Conversely, recent reports have demonstrated that mTOR, Rheb, and TSC2 colocalize with a lysosomal marker, called LAMP2 (lysosome-associated membrane protein 2) [46,55]. When the TSC-TBC complex is phosphorylated by upstream regulators, it dissociates from the lysosome, preventing TSC2 from hydrolyzing Rheb-GTP on the lysosome, although the GAP activity of TSC2 remains [46,56,57]. A recent study noted that insulin stimulates acute dissociation of the TSC-TBC complex from the lysosomal surface, on which subpopulations of Rheb and mTORC1 reside, showing that Akt-mediated phosphorylation of TSC2 effects the dissociation of the TSC-TBC complex from the lysosome in response to insulin [58]. As discussed, phosphorylation of the TSC-TBC complex activates Rheb and mTORC1 signaling. TSC-TBC and Rheb have a significant effect on the activation of mTORC1, in what we refer to as the Rhebulator-Rheb model, shown in Figure 1.

Rheb needs a guanine exchange factor (GEF) to transition from its GDP-bound to GTP-bound form [59], but GEF for Rheb has not been determined. Although Hsu *et al.* [60,61,62,63] suggested that Drosophila translationally controlled tumor protein (dTCTP) is a GEF of dRheb and that TCTP is essential for growth and proliferation through regulation of the dRhebGTPase, other groups have reported that deletion and overexpression of TCTP have no effect on mTORC1 activity and that TCTP does not have GTP exchange activity toward Rheb in mammalian cells. Recent studies have demonstrated that E3 ubiquitin ligase associates with Myc (PAM) and activate Rheb directly as a GEF and that deacetylated αβ-tubulin increases the GTP-loading of Rheb as a positive regulator [64,65]. However, these findings must be validated. 

## 3. Models of mTORC1 Activation by Amino Acids

### 3.1. Ragulator-Rags Model

Ragulator is a pentameric complex in which LAMTOR4 and LAMTOR5 form a heterodimer that interacts with the MP1-p14 heterodimer through p18 on the lysosomal membrane [66,67]. Ragulator is believed to have GEF activity toward RagA and RagB, which belong to the Ras-related GTPase (Rag GTPase) family. There are four mammalian Rags: RagA, RagB, RagC, and RagD [68,69,70]. GTP-bound RagA/B and GDP-bound RagC/D are central to the amino acid-sensitive mTORC1 pathway [69,71,72]. 

Rag GTPases localize to the lysosomal membrane to recruit mTORC1 by interacting with Raptor [66,71]. The activated GTP bound-RagA/B and GDP bound-RagC/D Rag GTPases can activate mTORC1, but it is unknown how they are converted from the GDP-bound and GTP-bound forms, respectively. Minimally, these processes require GEFs and GAPs. Recent findings have demonstrated Ragulator to be a GEF for RagA/B, and FLCN (also known as folliculin) and its binding partner, FNIP1/2 (FLCN-interacting protein 1/2), are Rag-binding proteins with GAP activity for RagC/D [67,73]. Also, leucyl-tRNA synthetase (LRS) is a GAP for RagD only [74,75]. 

SH3 domain-binding protein 4 (SH3BP4), Gator1, and the adaptor protein p62 are key factors in the mTORC1 signaling pathway. SH3BP4 interacts with inactive Rags through its Src homology 3 (SH3) domain under amino acid starvation to impede the formation of an active Rag GTPase complex, and Gator1 acts as a GAP, promoting transition of the active GTP-bound RagA/B to its inactive GDP-bound form, which inhibits mTORC1 activation [76,77,78,79,80,81]. In contrast, p62 binds to Rag proteins to favor the formation and translocation of active Rag GTPases, and it is a positive regulator of Rag GTPases [82]. 

In summary, amino acids enter the cell through specific channels and promote the GEF activity of Ragulator and the GAP activity of FLCN-FNIP1/2 and LRS to stimulate Rag GTPase. Subsequently, the activated Rags recruit mTORC1 to the lysosome to interact with Rheb-GTP, initiating mTORC1 signaling [66,67,71,73,74,75,83,84,85]. 

**Figure 2:**
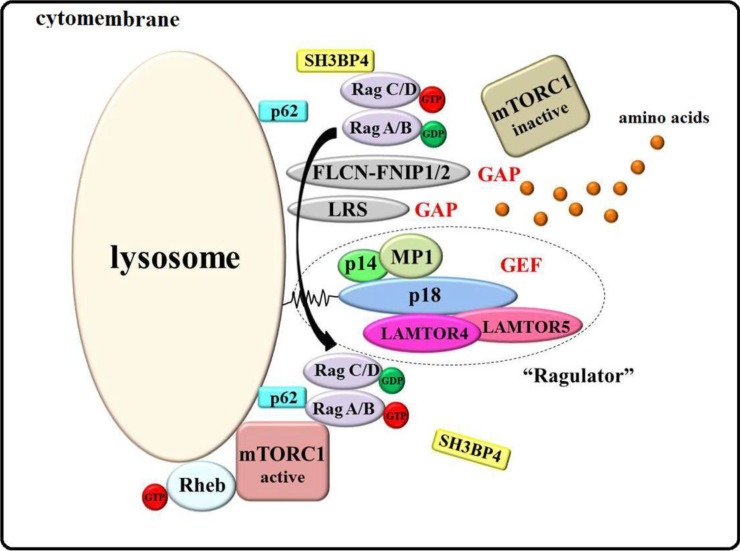
The Ragulator-Rags model of mTORC1 activation by amino acids. Ragulator, a GEF, comprises p14, MP1, p18, LAMTOR4, and LAMTOR5, which localize to the lysosomal surface through p18. Under sufficient amino acids, Ragulator recruits Rag GTPases to the lysosome. Concurrently, amino acids promote the GEF activity of Ragulator for GDP-bound RagA/B and the GAP activity of FLCN-FNIP1/2 and LRS for GTP-bound RagC/D, and active Rag GTPases can recruit mTORC1 to the lysosome to interact with GTP-bound Rheb, which localizes to lysosome, initiating mTORC1 signaling. SH3BP5 is a negative regulator of Rags that interacts with inactive Rag GTPases under amino acid starvation to impede the formation of an active Rag GTPase complex, which inhibits mTORC1 activation. In contrast, p62 binds to Rag proteins to favor the formation and localization of active Rag GTPases, which regulate mTORC1.

Menon *et al.* [58] and Demetriades *et al.* [86] have provided additional insight into the regulation of mTORC1 by amino acids. When cells have sufficient amino acids, TORC1 is active, due to its lysosomal relocalization, mediated by Rag GTPases. Upon depletion of amino acids, Rag GTPases release TORC1, causing it to become cytoplasmic and inactive [58,86]. Although this model comprises many unknown mechanisms, it provides an overall framework of mTORC1 activation by amino acids, which we have named the Ragulator-Rags model, shown in Figure 2. 

### 3.2. Amino Acids “inside-out” Model

The mechanism through which amino acid signals are sensed by mTORC1 remains unknown. Recent findings show that amino acids initiate signals within the lysosome, constituting the “inside-out” mechanism. In the egress of amino acids from the lysosome, vacuolar H^+^-adenosine triphosphatase (v-ATPase) resides on the lysosome, recruiting Rag GTPases by hydrolyzing ATP [83,84,87,88]. The amino acids within the lysosome induce structural rearrangement of the v-ATPase-Ragulator-Rag GTPase [87]. However, the factor that senses amino acids is unknown. 

As early as 2001, some findings demonstrated that lysosome amino acid transporter 1 (LYAAT1), also known as proton-coupled amino acid transporter 1 (PAT1), actively exports amino acids from lysosomes by chemiosmotic coupling to v-ATPase on the lysosomal membrane [89]. Recent research has shown that PAT1 is required for mTORC1 activation by amino acids, exporting amino acids from the lysosomal lumen to the cytosol to activate mTORC1 during this process [84,89,90,91,92,93]. PAT1 senses the changes in amino acid concentrations in the lysosomal lumen and exports amino acids. This process induces rearrangement of the v-ATPase-Ragulator-Rag GTPase and colocalization of mTORC1 with Rag GTPase at the lysosome and its interaction with Rheb-GTP. 

**Figure 3:**
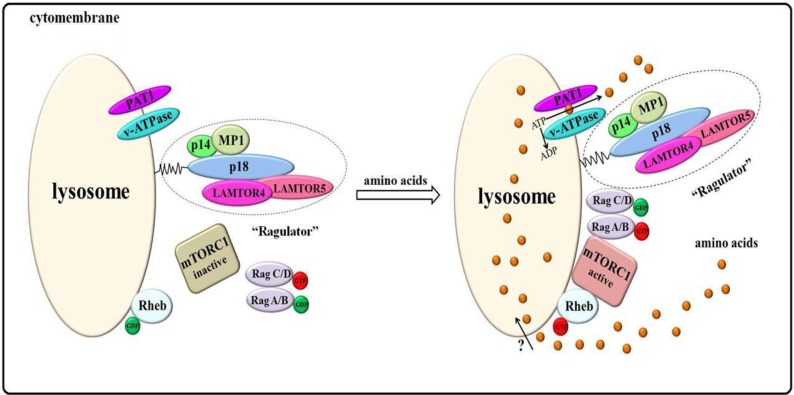
The “inside-out” model of mTORC1 activation by amino acids. Ragulator, PAT1, v-ATPase, and Rheb localize on the lysosomal surface. The amino acid signals in the lysosome are sensed by v-ATPase and PAT1, which actively export amino acids from the lumen to the cytosol and induce structural rearrangement of the v-ATPase-Ragulator-Rag GTPase by hydrolyzing ATP. This process activates the Ragulator and Rag GTPases, which colocalize mTORC1 to the lysosome to interact with active Rheb-GTP on the surface, initiating mTORC1 signaling.

We refer to this model as the amino acids “inside-out” model, as shown in Figure 3. This process is initiated in lysosomes, for which PAT1 is an amino acid sensor and v-ATPase is a key factor. Nevertheless, several mechanisms in this model remain unknown. For example, the transporter that amino acids use to enter lysosomes.

### 3.3. The “hVps34/Rheb-PLD1” Model

Human vacuolar protein sorting 34 (hVps34), the sole class III member of the PI3 Kinase family, participates in mTORC1 activation by amino acids [94,95,96,97]. Gulati *et al.* [98] provided evidence that stimulation by amino acids raises intracellular Ca^2+^ levels, which enhances the interaction between Ca^2+^/CaM and hVps34, resulting in hVps34 activation. However, another group believes that hVps34 activity is regulated through its interactions with human vacuolar protein sorting 15 (hVps15) and is independent of Ca^2+^/CaM [99]. 

Nevertheless, activated hVps34 is an important kinase that catalyzes phosphatidylinositol (PI) phosphorylation to form phosphatidylinositol 3-phosphate (PI3P). PI3P binds to the PX domain of phospholipase D1 (PLD1) to activate it [100,101,102,103]. Activated PLD1 hydrolyzes phosphatidylcholine (PC) to produce phosphatidic acid (PA). PA then competes with rapamycin/FKBP12, binding to the FRB domain of mTOR to promote mTORC1 signaling [18,104,105,106,107]. 

Recently, the crystal structures of mTOR kinase and mTORC1 were solved, demonstrating that the binding of rapamycin/FKBP12 to mTORC1 restricts substrate access to the active site, such as S6K1, and causes the disintegration of mTORC1 to abolish the phosphorylation of 4E-BP1 [2,108]. New evidence indicates that Rheb binds and activates PLD1 in a GTP-dependent manner, suggesting that PLD1 is an effector of Rheb in the mTORC1 pathway and is a positive regulator of mTORC1 activation by amino acids [109]. 

**Figure 4:**
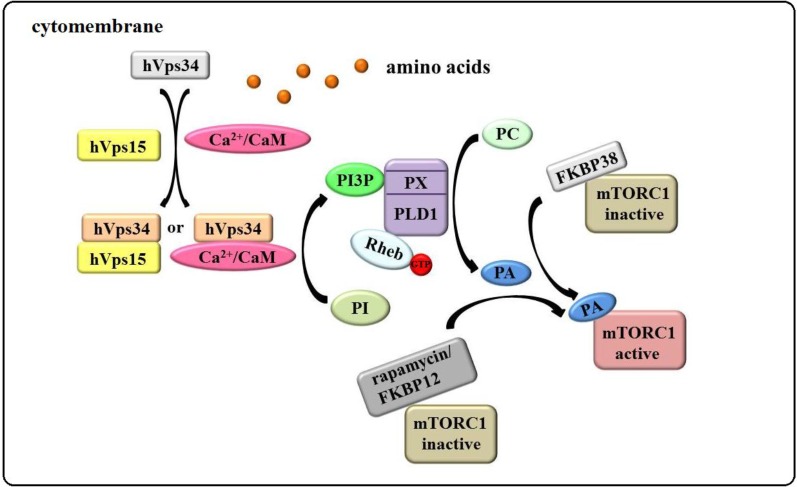
The“hVps34/Rheb-PLD1” model of mTORC1 activation by amino acids. Amino acids stimulate Ca^2+^/CaM or hVps15, interacting with hVps34 to activate it. The activated hVps34 complex catalyzes PI to produce PI3P, which associates with the PX domain of PLD1 to stimulate PLD1. Concurrently, active GTP-bound Rheb that is stimulated by amino acids also promotes PLD1 activity. Subsequently, the product of phosphatidylcholine (PC) hydrolysis by PLD1, PA, binds directly to the FRB domain of mTOR and competitively inhibits rapamycin-FKBP12 to complex with mTOR. This process can activate mTORC1 directly.

Based on these results, we proposed the “hVps34/Rheb-PLD1” model, in which amino acids stimulate the interaction between Ca^2+^/CaM and hVps34 or between hVps15 and hVps34 to activate hVps34. Then, the product of hVps34, PI3P, interacts with the PX domain of PLD1 to promote its activity. Subsequently, the product of phosphatidylcholine hydrolysis by PLD1, PA, binds directly to the FRB domain of mTOR and competitively inhibits rapamycin-FKBP12 to complex with mTORC1 (Figure 4).

### 3.4. Rheb Binds to FKBP38 and mTOR Is Unleashed

**Figure 5:**
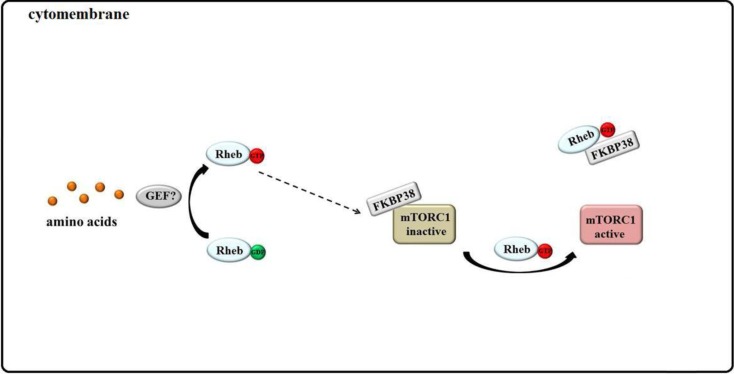
Model of mTORC1 activation by amino acids: “Rheb binds to FKBP38 and mTOR is unleashed.” Under conditions of ample amino acids, Rheb-GTP might interact with FKBP38 and induce the release and activation of mTOR.

As a unique member of the FKBP (FK506-binding protein) family, FKBP38 contains three types of domains: FKBP_C, TPR, and TM. In the absence of growth factors and nutrition, the FKBP_C domain can bind to mTOR to suppress the phosphorylation of S6K1 and 4EBP1 [53]. In the current model of the Rheb–FKBP38–mTOR relationship, FKBP38 is an endogenous inhibitor of mTOR. Under amino acid or serum starvation, this mTOR inhibitor binds to and interferes with mTORC1 function, similar to the FKBP12-rapamycin complex. Under conditions of ample growth factors and nutrients, the FKBP_C domain might interact with Rheb-GTP to activate downstream mTOR signaling by releasing mTOR from FKBP38 [53,110] (Figure 5).

Activation of mTORC1 could be impaired by FKBP38, and FKBP38*#x2013;Rheb interactions have been confirmed experimentally [53,111], but other data do not support this model. Overexpression or knockdown of FKBP38 does not affect the phosphorylation of mTORC1 substrates [112,113]. Subsequent studies have shown that FKBP38 is not involved in the Rheb-dependent activation of mTORC1 *in vitro* [114], and the interaction between Rheb and FKBP38 could not be detected by three separate *in vitro* assays [115]. It remains unknown how Rheb-GTP activates mTORC1 and how FKBP38 inhibits mTORC1 signaling.

## 4. Conclusions

mTOR signaling is critical in many processes and human diseases, but it remains poorly characterized. Although we have developed basic models of mTORC1 activation by growth factors and amino acids, certain mechanisms in mTORC1 signaling are unknown. For example, how inositol polyphosphate multikinase (IPMK) and mitogen-activated protein 4 kinase 3 (MAP4K3) positively regulate amino acid-induced mTORC1 signaling. However, we do not know in which models these molecules participate or whether they constitute a new model. As a core signaling pathway, mTORC1 is regulated by many upstream factors and pathways. It is likely that other models of mTORC1 activation by growth factors and amino acids exist. Thus, examining the mTORC1 signaling pathway further is paramount, and future research should focus on determining the GEF of Rheb, amino acid sensors, how amino acids enter lysosome, and the mechanisms of mTORC1 activation.

## Abbreviations

mTOR: mammalian target of rapamycin; S6K1: ribosomal protein S6 kinase 1; 4E-BP1: eukaryotic translation initiation factor 4E-binding protein 1; ULK1: unc-51 like autophagy activating kinase 1; IGFs: insulin-like growth factors; PI3K: phosphatidylinositol-4, 5-bisphosphate 3-kinase; AKT: protein kinase B; ERK1/2: extracellular signal-regulated kinase 1/2; RSK1: p90 ribosomal S6 kinase; TSC: tuberous sclerosis; TBC1D7: TBC1 domain family, member 7; GAP: GTPase-activating protein; GEF: guanine exchange factor; PAT1: proton-coupled amino acid transporter 1; hVps34: human vacuolar protein sorting 34; PLD1: phospholipase D 1; Rheb: Ras homolog enriched in brain; FKBP: FK506-binding protein.
